# Supplementary material for: Anti-Inflammatory Oxysterol, Oxy210, Inhibits Atherosclerosis in Hyperlipidemic Mice and Inflammatory Responses of Vascular Cells
Source: Cells. 2024 Sep 30;13(19):1632. doi: 10.3390/cells13191632 (PMC11475996; doi:10.3390/cells13191632)
Supplement: Supplementary file 1 [file cells-13-01632-s001.zip › cells-3202103-supplementary.pdf]

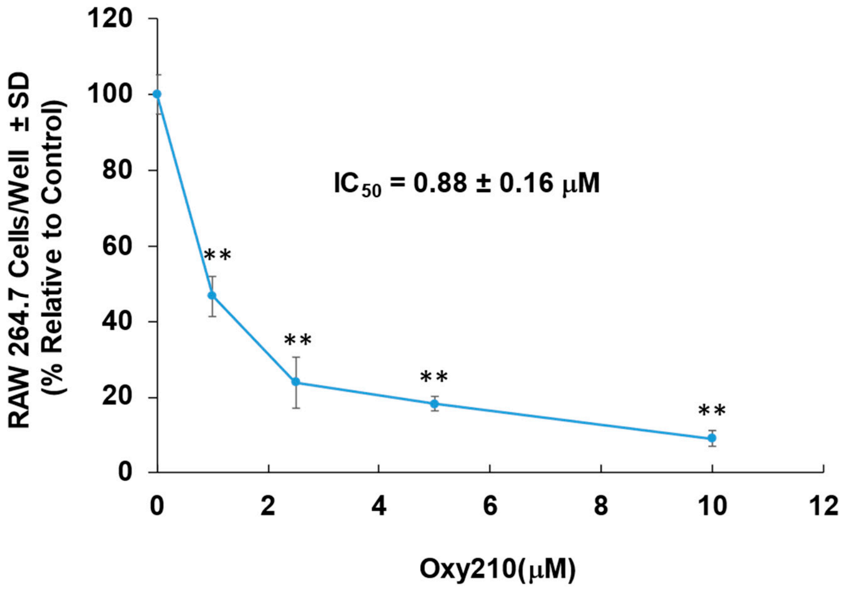

Supplementary Figure S1. Inhibition of RAW264.7 macrophage proliferation by Oxy210. RAW264.7 cells were treated with Oxy210 at increasing concentrations as indicated in DMEM containing 1% FBS for 48 hours, detached from wells and counted under a phase contrast microscope. Data from a representative experiment are reported as the mean of triplicate determinations ± SD (** $p < 0.01$ vs. untreated cells).

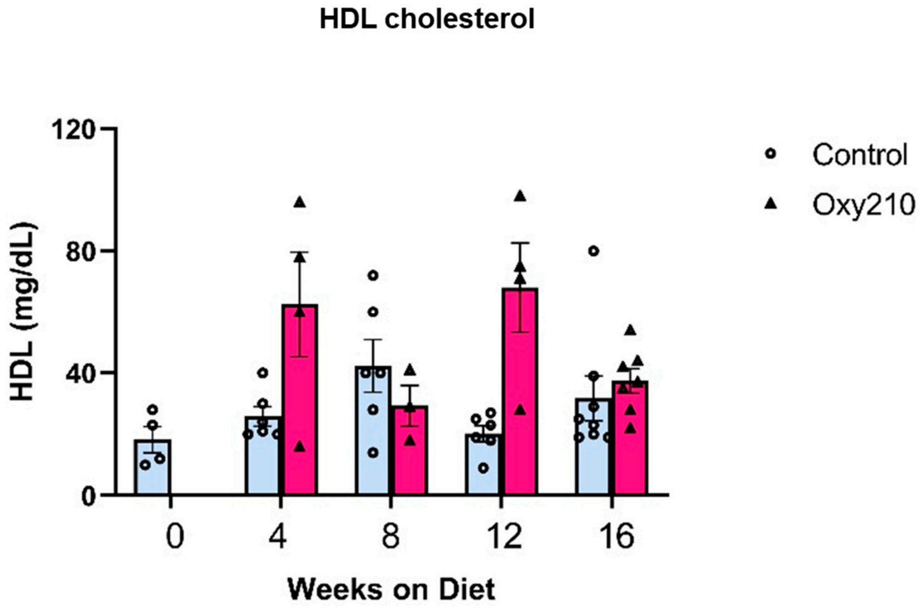

Supplementary Figure S2. Effect of Oxy210 on HDL levels. Female CETP/APOE*3-Leiden mice were fed Control WD without Oxy210 (blue bars) or with WD+Oxy210 (pinkbars) supplementation at 4mg/g diet for 0-16 weeks. Plasma was collected after

a 4-hr fast at the indicated time. Levels of HDL cholesterol were determined using a colorimetric assay. Results are presented as Mean + SEM from each group (n = 3-7).

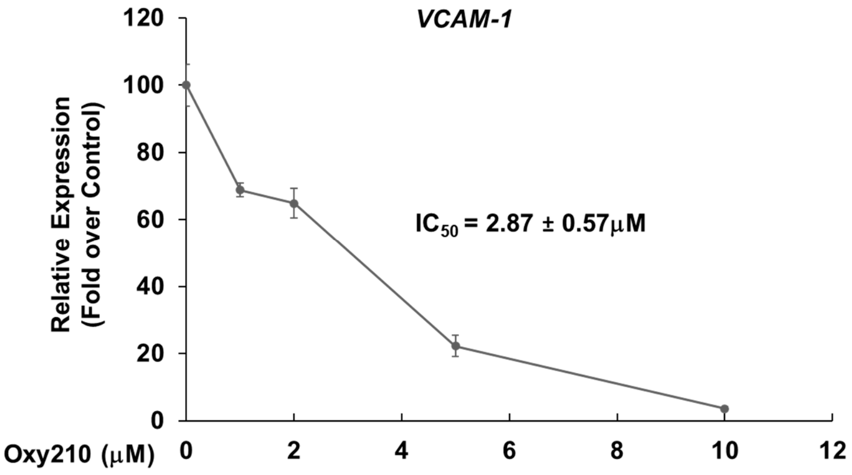

Supplementary Figure S3. Dose-dependent inhibition of LPS-induced *VCAM-1* expression in HAECs by Oxy210. HAECs were pretreated with increasing concentrations of Oxy210 as indicated in M199 containing 1% FBS overnight and then treated with 1 μg/ml of LPS in the absence or presence of Oxy210. After 4 hours, RNA was extracted and analyzed by Q-RT-PCR for the expression of the genes as indicated and normalized to GAPDH expression. Data from a representative experiment are reported as the mean of triplicate determinations ± SD (##p<0.01 vs. LPS; **p<0.01 vs control).

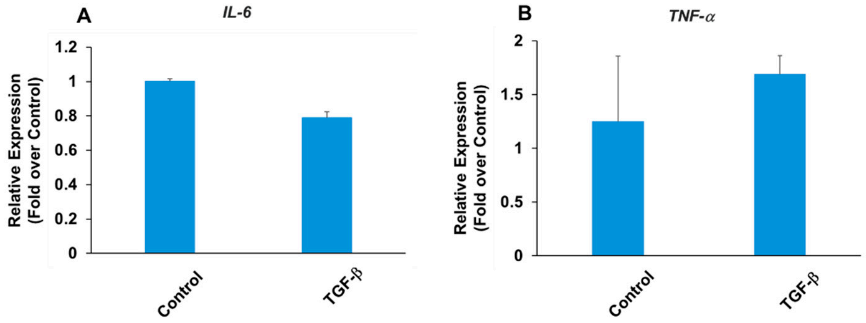

Supplementary Figure S4. TGF-β does not significantly induce *TNF-a* or *IL-6* expression in HAECs. HAECs in M199 containing 1% FBS were treated with rhTGF-β1 (10 ng/ml). After 48 hours, RNA was extracted and analyzed by Q-RT-PCR for the expression of the genes as indicated and normalized to GAPDH expression. Data from a representative experiment are reported as the mean of triplicate determinations ± SD.

Supplementary Table S1.

Mouse gene	Primers	Human gene	Primers
Oaz1	5′-CCACTGCTTCGCCAGAGAG-3′<br>5′-CCCGGACCCAGGTTACTA-3′	*GAPDH*	5′-CCTCAAGATCATCAGCAATGCCTCCT-3′<br>5′-GGTCATGAGTCCTTCCACGATACCAA-3′
Tnf–α	5′-CAGGCGGTGCCTATGTCTC-3′<br>5′-CGATCACCCCGAAGTTCAGTAG-3′	*VCAM-1*	5′-GGGAAGATGGTCGTGATCCTT-3′<br>5′-TCTGGGGTG GTCTCGATTTTA-3′
Il-1β	5′-GCAACTGTTCCTGAACTCAACT-3′<br>5′-ATCTTTTGGGGTCCGTCAACT-3′	*SELE*	5′-AGAGTGGAGCCTGGTCTTACA-3′<br>5′-CCTTTGCTGACAATAAGCACTGG-3′
JunB	5′-TCACGACGACTCTTACGCAG-3′<br>5′-CCTTGAGACCCCGATAGGGA-3′	*M-CSF*	5′-TGGCGAGCAGGAGTACAC-3′<br>5′-AGGTCTCCATCTGACTGTCAAT-3′
C-Src	5′-GAACCCGAGAGGGACCTTC-3′<br>5′-GAGGCAGTAGGCACCTTTTGT-3′	*TNF-α*	5′-GAGGCCAAGCCCTGGTATG-3′<br>5′-CGGGCCGATTGATCTCAGC-3′
Ccl2	5′-TTAAAAACCTGGATCGGAACCAA-3′<br>5′-GCATTAGCTTCAGATTTACGGGT-3′	*CCL2*	5′-CAGCCAGATGCAATCAATGCC-3′<br>5′-TGGAATCCTGAACCCACTTCT-3′
Il-6	5′-TAGTCCTTCCTACCCCAATTTCC-3′<br>5′-TTGGTCCTTAGCCACTCCTTC-3′	*MMP2*	5′-CCCACTGCGGTTTTCTCGAAT-3′<br>5′-CAAAGGGGTATCCATCGCCAT-3′
Rpl4	5′-GGAAGTTGGATGAGCTGTAT-3′<br>5′-TCAAGATTCTGCTAAGGTCG-3′		
